# Sex differences in the association between vitamin D and prediabetes in adults: A cross-sectional study

**DOI:** 10.1038/s41387-024-00311-4

**Published:** 2024-07-02

**Authors:** Ali H. Ziyab, Anwar Mohammad, Zainab Almousa, Talal Mohammad

**Affiliations:** 1 Department of Community Medicine and Behavioral Sciences, College of Medicine, Kuwait University, Safat, Kuwait; 2 Dasman Diabetes Institute, Biochemistry and Molecular Biology Department, Kuwait City, Kuwait; 3 St. Antony’s College, University of Oxford, Oxford, UK

**Keywords:** Pre-diabetes, Pre-diabetes

## Abstract

**Background/Objectives:**

Vitamin D status has been shown to be associated with prediabetes risk. However, epidemiologic evidence on whether sex modulates the association between vitamin D and prediabetes is limited. The present study investigated sex-specific associations between vitamin D and prediabetes.

**Subjects/Methods:**

The Kuwait Wellbeing Study, a population-based cross-sectional study, enrolled nondiabetic adults. Prediabetes was defined as 5.7 ≤ HbA1c% ≤6.4; 25-hydroxyvitamin D (25(OH)D) was measured in venous blood and analyzed as a continuous, dichotomous (deficiency: <50 nmol/L vs. insufficiency/sufficiency ≥50 nmol/L), and categorical (tertiles) variable. Associations were evaluated by estimating adjusted prevalence ratios (aPRs) and 95% confidence intervals (CIs), while stratifying by sex.

**Results:**

A total of 384 participants (214 males and 170 females) were included in the current analysis, with a median age of 40.5 (interquartile range: 33.0–48.0) years. The prevalence of prediabetes was 35.2%, and 63.0% of participants had vitamin D deficiency. Assessments of statistical interaction between sex and 25(OH)D status were statistically significant (*P*_Sex × 25(OH)D Interaction_ < 0.05). In the sex-stratified analysis, after adjustment for confounding factors, decreased 25(OH)D levels were associated with increased prevalence of prediabetes in males (aPR_Deficiency vs. In-/Sufficiency_: 2.35, 95% CI: 1.36–4.07), but not in females (aPR_Deficiency vs. In-/Sufficiency_: 1.03, 95% CI: 0.60–1.77). Moreover, the prevalence of prediabetes differed between males and females at 25(OH)D levels of ≤35 nmol/L, with a higher prevalence of prediabetes in males compared to females. Such a sex-specific difference was not observed at 25(OH)D levels of >35 nmol/L.

**Conclusions:**

Sex modified the association between vitamin D levels and prediabetes, with an inverse association observed among males, but not among females. Moreover, the observed sex-disparity in the prevalence of prediabetes was only pronounced at 25(OH)D levels of ≤35 nmol/L.

## Introduction

Prediabetes is characterized by higher than normal glycemic levels but lower than the diagnostic threshold for diabetes [1]. The global prevalence of prediabetes, measured as impaired glucose tolerance (IGT), among adults aged 20–79 years was estimated to be 9.1% in 2021 and is projected to increase to 10.0% in 2045 [2]. According to impaired fasting glucose (IFG), the global prevalence of prediabetes among adults aged 20–79 years was estimated to be 5.8% in 2021 and is projected to increase to 6.5% in 2045 [2]. The wide variability in the prevalence of prediabetes within and between countries can be explained to some extent by the heterogeneity of the applied diagnostic tests and criteria, and the characteristics of the populations being studied [3]. Nonetheless, the prevalence of prediabetes is increasing worldwide, and such an increase will add to the pool of diabetes prevalence.

The annual progression (incidence) rate from prediabetes to diabetes has been estimated to be around 5–10%, with variations in the progression rate due to population characteristics and methods of prediabetes ascertainment [1]. A meta-analysis estimated the range of the 5-year progression rate from prediabetes (defined as glycated hemoglobin (HbA1c) between 6.0% and 6.5%) to diabetes to be 25% to 50% [4]. Other investigations have corroborated that prediabetes, regardless of the used diagnostic criteria, increases the risk of diabetes [5–7]. Moreover, prediabetes has been linked to the development of microvascular complications, such as neuropathy, retinopathy, and nephropathy, and has also been associated with increased risk of macrovascular complications, such as cardiovascular disease, stroke, and peripheral vascular disease [1, 7–10].

Vitamin D (a prototypical secosteroid) is essential for maintaining a healthy mineralized skeleton through the regulation of calcium and phosphate homeostasis [11]. In addition, due to the ubiquitous expression of vitamin D receptors (VDR) in nearly all tissues and cells, vitamin D (active form, 1,25-dihydroxyvitamin D), through interaction with VDR, exerts numerous pleiotropic extra-skeletal effects that play a role in immune responses, cardiovascular events, cancer, and metabolic diseases [11, 12]. Observational studies, although slightly inconsistent, have generally reported inverse associations between serum 25-hydroxyvitamin D (25(OH)D) levels and diabetes and prediabetes, with reduced vitamin D levels being associated with increased risk of diabetes and prediabetes [13–18]. Biologically, vitamin D has been shown to influence insulin secretion and sensitivity thereby disrupting glucose homeostasis [19, 20].

Methods of vitamin D ascertainment, study design and population, and confounding factors could contribute to the observed inconsistencies in the associations between vitamin D and prediabetes/diabetes [21]. Similarly, not evaluating potential effect modification could also lead to biased estimates of association. Sex has been shown to differentially influence disease epidemiology, pathogenesis, clinical presentation, progression, and response to medications [22, 23]. Studies have reported that sex is a biological factor that has been shown to regulate the level and effect of vitamin D, and that vitamin D levels differ between males and females [24, 25]. Sex may also influence overall vitamin D levels in the body and modulate the body’s response to vitamin D supplementation for the prevention and treatment of various conditions, one of which is prediabetes [24]. Therefore, assessing and identifying sex differences in disease etiology is an essential step toward sex-specific precision medicine that can improve the health of males and females. Sex is emerging as an effect modifier of the association between vitamin D and multiple diseases, including diabetes and prediabetes, due to the possibility that sex hormones may affect the potency and bioavailability of vitamin D in a sex-specific manner [24, 26]. Hence, we hypothesized that sex modulates the association between vitamin D and prediabetes. Given the scarcity of studies evaluating sex differences in the association between vitamin D and prediabetes, we sought to determine whether the association between vitamin D and prediabetes differs according to sex in a population-based sample of adults without diabetes.

## Subjects and methods

### Study design, setting, and population

A population-based cross-sectional study, the Kuwait Wellbeing study, was conducted between November 2012 and October 2017. A total of 1240 Kuwaiti adults aged between 18 and 65 years with no prior history of diabetes were enrolled in the study [27, 28]. The exclusion criteria included pregnancy, diagnosis of diabetes, inability to walk unaided, psychosis, or terminal illness. Of the total enrolled study sample, 56 participants were excluded as they met the undiagnosed diabetes criteria (i.e., no prior history of doctor-diagnosed diabetes and measured HbA1c ≥ 6.5% [48 mmol/mol]). Hence, 95.5% (1184/1240) of the enrolled study participants satisfied our inclusion criteria. The analytical sample in the current report (*n* = 384) is based on a random subsample of the total enrolled subjects (*n* = 1184) who had an available 25(OH)D measurement. The protocol of the present study was approved by the Ethical Review Board of the Dasman Diabetes Institute, Safat, Kuwait (RA-01-2010). Written informed consent was obtained from study participants. The study was conducted in accordance with the principles and guidelines of the Declaration of Helsinki for medical research involving human subjects. All assessments were completed in-person at the Dasman Diabetes Institute.

### Biochemical analysis and prediabetes and vitamin D status

Venous blood samples were collected from participants and transported in temperature-controlled boxes for processing and biochemical analysis at the institutional clinical laboratory that implements strict national and international quality assurance measures. The clinical laboratory is CAP (College of American Pathologists) accredited and has received diamond-level status from Accreditation Canada. HbA1c was measured using a VariantT M device (BioRad, Hercules, CA, USA). Among participants free of diabetes and according to the American Diabetes Association 2022 guidelines [29], HbA1c levels between 5.7–6.4% (39–47 mmol/mol) were indicative of prediabetes.

Levels of 25(OH)D were measured in the serum with chemiluminescent immunoassay (CLIA) by DiaSorin LIAISON^®^ analyzer (DiaSorin Inc, MN, USA) using direct competitive immunoassay LIAISON^®^ 25(OH) Vitamin D TOTAL Assay. Vitamin D status was categorized according to levels of 25(OH)D as follows: severe deficiency <25.0 nmol/L (10 ng/mL); deficiency ≥25 to <50 nmol/L (≥10 to <20 ng/mL); insufficiency ≥50 to <75 nmol/L (≥20 to <30 ng/mL); and sufficiency ≥75 nmol/L (≥30 ng/mL) [30, 31].

### Measurements of adiposity

Anthropometric measurements were performed by trained project nurses. Weight was measured to the nearest 0.1 kg using a calibrated digital scale (TANITA model BC-418 MA, Tokyo, Japan). A wall-mounted stadiometer (SECA model 240, Birmingham, UK) was used to measure height to the nearest 0.1 cm. Height and weight were assessed in light clothing and without shoes, following the study’s standardized operating procedures. Body mass index (BMI) was calculated as weight in kilograms divided by height in meters squared (kg/m^2^), and was categorized as: underweight/normal weight (<25.0 kg/m^2^), overweight (25.0 to 29.9 kg/m^2^), and obese (≥30.0 kg/m^2^) [32].

Lunar iDXA (dual-energy X-ray absorptiometry; GE Healthcare, Bedford, UK) was used to obtain total body imaging. On a daily bases and throughout the study period, quality assurance and control measures were performed before using the equipment according to manufacturer’s standard procedures. Standard imaging and positioning protocols were followed by trained operators to acquire body scans of participants [33]. Estimates of visceral adipose tissue (VAT) mass (kg) were derived using the iDXA enCORE software (version 14.10.022; GE Healthcare). The software estimates the VAT content within the android region while automatically placing a quadrilateral box, i.e., the region outlined by the iliac crest, with a superior height equivalent to 20% of the distance from the top of the iliac crest to the base of the skull [34].

### Covariates

Data on demographic information and lifestyle factors were collected using study questionnaires. Cigarette smoking status was self-reported and was ascertained as never smoker, former smoker (did not smoke any cigarettes in the past 30 days), and current smoker (smoked at least one cigarette in the past 30 days). Moreover, the frequency of walking (a proxy measure of physical activity) was assessed by asking: “In the last 4 weeks, how many times did you walk for pleasure (not as a means of transport)?”, with the following answer options: none, 1 to 3 times in the last 4 weeks, 1 to 3 times a week, 4 or more times a week. Moreover, season of blood collection was categorized based on the month of the blood draw as follows: Winter (December to February), Spring (March to May), Summer (June to August), and Fall (September to November).

### Statistical analysis

SAS 9.4 (SAS Institute, Cary, NC, USA) was used for statistical analyses. A two-sided *p*-value < 0.05 was considered statistically significant. Study variables were described by calculating the frequencies and proportions of categorical variables and medians and interquartile ranges (IQR; 25th percentile and 75th percentile) of continuous variables. VAT mass (kg) and 25(OH)D levels (nmol/L) were categorized into tertiles (lower, middle, and higher tertile). To account for sex differences, tertiles of VAT mass and 25(OH)D were determined separately for males and females.

Univariable analyses were conducted to assess associations between participants’ characteristics and prediabetes prevalence using chi-squared (χ^2^) test. Moreover, univariable analyses to evaluate associations between 25(OH)D levels and personal attributes of the participants were performed by applying Wilcoxon rank sum test when comparing two groups, and Kruskal-Wallis test when comparing three or more groups. Subsequently, multivariable analyses were conducted to evaluate association between 25(OH)D levels and prediabetes prevalence by applying a modified Poisson regression with robust variance estimation using the GENMOD procedure in SAS 9.4 [35]. Adjusted prevalence ratios (aPRs) and their 95% confidence intervals (CIs) were estimated. In all multivariable regression models, statistical adjustments for the effects of the following variables were made: age, BMI, smoking status, walking frequency, and season of blood collection. The 25(OH)D status variable was modeled as a: (i) continuous variable (per 10-unit decrease), (ii) dichotomous variable (deficiency vs. insufficiency/sufficiency [reference group]), and (iii) categorical variable using tertiles (lower, middle, and higher tertile [reference group]). To assess whether the association between 25(OH)D and prediabetes differs according to sex (effect modification by sex), statistical interactions on a multiplicative scale were evaluated by including a product term (sex × 25(OH)D) in the regression models. Subsequently, sex-stratified associations between 25(OH)D status and prediabetes prevalence were evaluated. Moreover, adjusted associations between sex (males vs. females) and prediabetes prevalence were assessed at different levels of 25(OH)D. Predicted probabilities of prediabetes were estimated for male and female participants across 25(OH)D levels using multivariable logistic regression.

### Sensitivity analysis

Given that adiposity has been shown to be a major confounder of association estimates relating vitamin D (25(OH)D levels) and disease risk [36], we have repeated our multivariable analyses while adjusting for VAT mass (measure of central adiposity; objectively assessed using iDXA) instead of BMI (measure of general adiposity). This allowed us to confirm the robustness of our primary analyses.

## Results

The characteristics of the total sample (*n* = 1184) and the analytical sample (*n* = 384) are shown in the online supplementary Table S1. The analytical sample and the total sample were similar with respect to all characteristics under study (Table S1). In the total analytical sample, the median age was 40.5 (IQR: 33.0–48.0) years, 55.7% of subjects were males, 35.2% had prediabetes, and 34.9% and 28.1% were classified to suffer from severe vitamin D deficiency (25(OH)D < 25 nmol/L) and vitamin D deficiency (25 ≤ 25(OH)D < 50 nmol/L), respectively (Table 1). Cigarette smoking, prediabetes, overweight/obesity, increased VAT, and vitamin D deficiency were more common in males than females (Table 1).

**Table 1:** Characteristics of total analytical sample and stratified by sex.

Variables	Total (*n* = 384)	Males (*n* = 214)	Females (*n* = 170)	*P*-value
**Sex, % (*n*)**				
Male	55.7 (214)	–	–	–
Female	44.3 (170)	–	–	–
**Age (years), % (*n*)**				
Overall, Median (IQR)	40.5 (33.0–48.0)	40.0 (31.0–48.0)	41.0 (34.0–48.0)	0.505^f^
≤34	30.5 (117)	34.6 (74)	25.3 (43)	0.165^e^
35–44	32.0 (123)	28.0 (60)	37.0 (63)	
45–54	27.1 (104)	27.1 (58)	27.1 (46)	
≥55	10.4 (40)	10.3 (22)	10.6 (18)	
**Cigarette smoking status, % (*n*)**				
Never smoker	54.5 (180)	29.7 (55)	86.2 (125)	<0.001^e^
Former smoker	17.6 (58)	28.7 (53)	3.5 (5)	
Current smoker	27.9 (92)	41.6 (77)	10.3 (15)	
Missing, (*n*)	(54)	(29)	(25)	
**Walking in the past 4 weeks, % (*n*)**				
None	29.5 (100)	27.4 (52)	32.2 (48)	0.590^e^
1 to 3 times in the last 4 weeks	36.0 (122)	36.3 (69)	35.6 (53)	
1 to 3 times a week	21.8 (74)	24.2 (46)	18.8 (28)	
4 or more times a week	12.7 (43)	12.1 (23)	13.4 (20)	
Missing, (*n*)	(45)	(24)	(21)	
**Prediabetes (HbA1c), % (*n*)**				
Yes (5.7–6.4%)	35.2 (135)	39.4 (84)	30.0 (51)	0.055^e^
No (<5.7%)	64.8 (248)	60.6 (129)	70.0 (119)	
Missing, (*n*)	(1)	(1)	(0)	
**BMI (kg/m**^**2**^**), % (*n*)**				
Underweight/normal weight (<25.0)^a^	21.4 (82)	17.3 (37)	26.5 (45)	0.088^e^
Overweight (25.0 to <30.0)	45.6 (175)	48.6 (104)	41.7 (71)	
Obesity (≥30.0)	33.0 (127)	34.1 (73)	31.8 (54)	
**VAT (kg), Median (IQR)**				
Overall (*n* = 384^b^, 214^c^, 170^d^)	0.89 (0.45–1.51)	1.29 (0.66–1.86)	0.64 (0.32–0.95)	<0.001^f^
Tertile 1 (*n* = 137^b^, 74^c^, 63^d^)	0.31 (0.18–0.49)	0.46 (0.24–0.71)	0.22 (0.11–0.36)	
Tertile 2 (*n* = 136^b^, 75^c^, 61^d^)	1.08 (0.72–1.44)	1.41 (1.19–1.55)	0.69 (0.59–0.78)	
Tertile 3 (*n* = 111^b^, 65^c^, 46^d^)	1.87 (1.32–2.29)	2.19 (1.92–2.47)	1.28 (1.04–1.42)	
**25-hydroxyvitamine D (nmol/L), % (n)**				
Severe deficiency (<25)	34.9 (134)	38.3 (82)	30.6 (52)	<0.001^e^
Deficiency (≥25 to <50)	28.1 (108)	39.2 (84)	14.1 (24)	
Insufficiency (≥50 to <75)	24.7 (95)	16.4 (35)	35.3 (60)	
Sufficiency (≥75)	12.3 (47)	6.1 (13)	20.0 (34)	
**25-hydroxyvitamine D (nmol/L), Median (IQR)**				
Overall (*n* = 384^b^, 214^c^, 170^d^)	32.0 (20.0–62.0)	27.0 (21.0–46.0)	55.0 (20.0–70.0)	<0.001^f^
Tertile 1 (*n* = 123^b^, 66^c^, 57^d^)	16.0 (14.0–20.0)	17.0 (14.0–20.0)	15.0 (13.0–20.0)	
Tertile 2 (*n* = 137^b^, 77^c^, 60^d^)	32.0 (26.0–55.0)	27.0 (25.0–30.0)	56.0 (45.0–64.0)	
Tertile 3 (*n* = 124^b^, 71^c^, 53^d^)	69.0 (53.5–82.5)	55.0 (46.0–67.0)	79.0 (73.0–95.0)	

*IQR* interquartile range, *HbA1c* glycated hemoglobin, *BMI* body mass index, *VAT* visceral adipose tissue.

^a^The underweight group (BMI < 18.5 kg/m^2^) was analyzed with the normal weight group due to only one subject (1/384, 0.3%) being underweight.

Number of participants in: ^b^total analytical sample, ^c^males, and ^d^females.

^e^Calculated using chi-square test.

^f^Calculated using the Wilcoxon rank sum test.

Table 2 shows the prevalence of prediabetes according to personal characteristics in the total analytical sample and stratified by sex. The prevalence of prediabetes was higher in male compared to female participants (39.4% vs. 30.0%, *P* = 0.055). In both males and females, the prevalence of prediabetes increased as age increased. Increased BMI and VAT mass were associated with an increased prevalence of prediabetes. Among male participants, the prevalence of prediabetes was higher among subjects with deficient levels of vitamin D compared to subjects with insufficient/sufficient vitamin D levels (43.0% vs. 27.1, *P* = 0.047). However, among females, the prevalence of prediabetes tended to be higher among participants with insufficient/sufficient vitamin D levels compared to participants with deficient levels of vitamin D (36.2% vs. 22.4, *P* = 0.051; Table 2).

**Table 2:** Prevalence of prediabetes according to personal characteristics in the total analytical sample and stratified by sex.

	Prevalence of prediabetes, % (*n*/total)		
Variables	Total analytical sample	Males	Females
**Sex**			
Male	39.4 (84/213)	–	–
Female	30.0 (51/170)	–	–
* P*-value^*^	0.055	–	–
**Age (years)**			
≤34	21.4 (25/117)	24.3 (18/74)	16.3 (7/43)
35–44	27.6 (34/123)	40.0 (24/60)	15.9 (10/63)
45–54	48.5 (50/103)	50.9 (29/57)	45.7 (21/46)
≥55	65.0 (26/40)	59.1 (13/22)	72.2 (13/18)
* P*-value^*^	<0.001	0.003	<0.001
**Cigarette smoking status**			
Never smoker	37.8 (68/180)	45.5 (25/55)	34.4 (43/125)
Former smoker	44.8 (26/58)	47.2 (25/53)	20.0 (1/5)
Current smoker	28.6 (26/91)	32.9 (25/76)	6.7 (1/15)
*P*-value^*^	0.115	0.187	0.078
**Walking in the past 4 weeks**			
None	41.0 (41/100)	40.4 (21/52)	41.7 (20/48)
1 to 3 times in the last 4 weeks	34.7 (42/121)	41.2 (28/68)	26.4 (14/53)
1 to 3 times a week	33.8 (25/74)	37.0 (17/46)	28.6 (8/28)
4 or more times a week	30.2 (13/43)	34.8 (8/23)	25.0 (5/20)
*P*-value^*^	0.583	0.935	0.329
**BMI (kg/m**^**2**^**)**			
Underweight/normal weight (<25.0)^a^	22.0 (18/82)	24.3 (9/37)	20.0 (9/45)
Overweight (25.0 to <30.0)	36.6 (64/175)	39.4 (41/104)	32.4 (23/71)
Obesity (≥30.0)	42.1 (53/126)	47.2 (34/72)	35.2 (19/54)
*P*-value^*^	0.011	0.068	0.220
**VAT (kg)**			
Tertile 1	18.3 (25/137)	17.6 (13/74)	19.1 (12/63)
Tertile 2	42.7 (58/136)	48.0 (36/75)	36.1 (22/61)
Tertile 3	47.3 (52/110)	54.7 (35/64)	37.0 (17/46)
*P*-value^*^	<0.001	<0.001	0.057
**25-hydroxyvitamine D (nmol/L)**			
Severe deficiency (<25)	38.1 (51/134)	48.8 (40/82)	21.2 (11/52)
Deficiency (≥25 to <50)	34.6 (37/107)	37.4 (31/83)	25.0 (6/24)
Insufficiency (≥50 to <75)	37.9 (36/95)	31.4 (11/35)	41.7 (25/60)
Sufficiency (≥75)	23.4 (11/47)	15.4 (2/13)	26.5 (9/34)
*P*-value^*^	0.299	0.065	0.097
Deficiency (<50)	36.5 (88/241)	43.0 (71/165)	22.4 (17/76)
In-/Sufficiency (≥50)	33.1 (47/142)	27.1 (13/48)	36.2 (34/94)
*P*-value^*^	0.499	0.047	0.051
Tertile 1	34.2 (42/123)	47.0 (31/66)	19.3 (11/57)
Tertile 2	40.4 (55/136)	42.1 (32/76)	38.3 (23/60)
Tertile 3	30.7 (38/124)	29.6 (21/71)	32.1 (17/53)
*P*-value^*^	0.244	0.096	0.074

*HbA1c* glycated hemoglobin, *BMI* body mass index, *VAT* visceral adipose tissue.

^a^The underweight group (BMI < 18.5 kg/m^2^) was analyzed with the normal weight group due to only one subject (1/384, 0.3%) being underweight.

^*^Calculated using chi-square test.

Table 3 shows the levels of vitamin D (25(OH)D) according to personal characteristics in the total analytical sample and stratified by sex. The median vitamin D levels were higher in female compared to male participants (55.0 vs. 27.0 nmol/L, *P* < 0.001). Among male participants, vitamin D levels differed according to prediabetes status, with higher vitamin D levels observed among participants with no prediabetes compared to those with prediabetes (29.0 vs. 25.0 nmol/L, *P* = 0.025). Moreover, vitamin D levels tended to decrease as BMI increased among male participants. Among female participants, vitamin D levels increased as age increased, and vitamin D levels were influenced by smoking status, with current smokers having the lowest vitamin D levels. Vitamin D levels did not differ according to prediabetes status among female participants (no prediabetes: 51.0 vs. prediabetes: 58.0 nmol/L, *P* = 0.185; Table 3).

**Table 3:** Levels of 25-hydroxyvitamin D according to participants characteristics in the total analytical sample and stratified by sex.

	25-hydroxyvitamin D levels (nmol/L), Median (IQR)		
Variables	Total analytical sample	Males	Females
**Sex**			
Male (*n* = 214^a^)	27.0 (21.0–46.0)	–	–
Female (*n* = 170^a^)	55.0 (20.0–70.0)	–	–
*P*-value^*^	<0.001	–	–
**Age (years)**			
≤34 (*n* = 117^a^, 74^b^, 43^c^)	26.0 (19.0–42.0)	26.0 (20.0–35.0)	29.0 (15.0–69.0)
35–44 (*n* = 123^a^, 60^b^, 63^c^)	30.0 (17.0-62.0)	27.0 (19.0–53.0)	45.0 (15.0–66.0)
45–54 (*n* = 104^a^, 58^b^, 46^c^)	47.0 (24.0–66.5)	27.5 (22.0–53.0)	66.0 (48.0–79.0)
≥55 (*n* = 40^a^, 22^b^, 18^c^)	55.5 (28.5–67.5)	33.0 (25.0–61.0)	62.5 (56.0–74.0)
*P*-value^*^	<0.001	0.235	<0.001
**Cigarette smoking status**			
Never smoker (*n* = 180^a^, 55^b^, 125^c^)	45.0 (22.0–66.0)	29.0 (22.0–43.0)	56.0 (24.0–70.0)
Former smoker (*n* = 58^a^, 53^b^, 5^c^)	30.0 (20.0–56.0)	30.0 (22.0–54.0)	64.0 (17.0–79.0)
Current smoker (*n* = 92^a^, 77^b^, 15^c^)	25.0 (18.0–38.0)	25.0 (20.0–38.0)	18.0 (12.0–56.0)
*P*-value^*^	<0.001	0.209	0.028
**Walking in the past 4 weeks**			
None (*n* = 100^a^, 52^b^, 46^c^)	36.5 (21.0–62.0)	28.0 (21.5–41.0)	55.5 (20.5–68.5)
1 to 3 times in the last 4 weeks (*n* = 122^a^, 69^b^, 53^c^)	28.5 (18.0–61.0)	25.0 (19.0–48.0)	54.0 (18.0–70.0)
1 to 3 times a week (*n* = 74^a^, 46^b^, 28^c^)	33.0 (23.0–55.0)	28.0 (22.0–40.0)	54.0 (30.5–64.0)
4 or more times a week (*n* = 43^a^, 23^b^, 20^c^)	37.0 (22.0–69.0)	34.0 (25.0–52.0)	67.5 (18.5–88.0)
*P*-value^*^	0.581	0.556	0.605
**Prediabetes (HbA1c)**			
Yes (5.7–6.4%) (*n* = 135^a^, 84^b^, 51^c^)	30.0 (20.0–62.0)	25.0 (19.0–35.0)	58.0 (39.0–72.0)
No (<5.7%) (*n* = 248^a^, 129^b^, 119^c^)	34.0 (20.0–62.0)	29.0 (22.0–50.0)	51.0 (18.0–70.0)
*P*-value^*^	0.576	0.025	0.185
**BMI (kg/m**^**2**^**)**			
Underweight/normal weight (<25.0) (*n* = 82^a^, 37^b^, 45^c^)	37.5 (21.0–65.0)	31.0 (22.0–46.0)	57.0 (21.0–69.0)
Overweight (25.0 to <30.0) (*n* = 175^a^, 104^b^, 71^c^)	31.0 (21.0–61.0)	27.0 (23.5–47.5)	55.0 (15.0–74.0)
Obesity (≥30.0) (*n* = 127^a^, 73^b^, 54^c^)	28.0 (18.0–61.0)	25.0 (18.0–36.0)	54.0 (20.0–66.0)
*P*-value^*^	0.236	0.089	0.618
**VAT (kg)**			
Tertile 1 (*n* = 137^a^, 74^b^, 63^c^)	34.0 (22.0–57.0)	30.0 (24.0–47.0)	47.0 (20.0–70.0)
Tertile 2 (*n* = 136^a^, 75^b^, 61^c^)	30.0 (18.0–64.0)	27.0 (19.0–46.0)	56.0 (16.0–73.0)
Tertile 3 (*n* = 111^a^, 65^b^, 46^c^)	35.0 (21.0–62.0)	25.0 (19.0–40.0)	59.5 (39.0–70.0)
*P*-value^*^	0.602	0.139	0.472
**Season of blood collection**			
Winter (December to February) (*n* = 142^a^, 76^b^, 66^c^)	29.0 (21.0–63.0)	25.0 (21.0–35.0)	62.5 (21.0–74.0)
Spring (March to May) (*n* = 122^a^, 64^b^, 58^c^)	38.5 (22.0–64.0)	32.0 (22.0–53.5)	51.5 (20.0–67.0)
Summer (June to August) (*n* = 72^a^, 44^b^, 28^c^)	28.0 (18.0–63.5)	27.0 (19.5–58.5)	39.5 (16.0–72.5)
Fall (September to November) (*n* = 48^a^, 30^b^, 18^c^)	30.0 (18.0–54.5)	29.5 (20.0–45.0)	46.5 (18.0–56.0)
*P*-value^*^	0.627	0.329	0.168

*IQR* interquartile range, *HbA1c* glycated hemoglobin, *BMI* body mass index, *VAT* visceral adipose tissue.

^*^Calculated using the Wilcoxon rank sum test when comparing the medians of two groups, and the Kruskal–Wallis test when comparing medians of three or more groups.

Number of participants in: ^a^ total analytical sample, ^b^ males, and ^c^ females.

Sex-stratified associations between vitamin D levels and prevalence of prediabetes were assessed by testing three modeling strategies, after adjusting for age, BMI, smoking status, walking frequency, and season of blood collection (Table 4). In model 1, the interaction term between sex and vitamin D levels (modeled as a continuous variable) was statistically significant (*P*_interaction_ = 0.032). Among male participants, the prevalence of prediabetes increased as vitamin D levels decreased (aPR_per 10-unit decrease in vitamin D_ = 1.19, 95% CI: 1.06–1.34). Such an association was not present among female participants (aPR_per 10-unit decrease in vitamin D_ = 1.02, 95% CI: 0.94–1.11). In model 2, we tested an interaction term between sex and a dichotomous vitamin D variable (insufficiency/sufficiency vs. deficiency), which was statistically significant (*P*_interaction_ = 0.026). In this model, vitamin D deficiency compared to insufficiency/sufficiency was associated with increased prevalence of prediabetes among male participants (aPR = 2.35, 95% CI: 1.36–4.07), but not among female participants (aPR = 1.03, 95% CI: 0.60–1.77; Table 4). In model 3, the sex-vitamin D tertiles interaction term was statistically significant (*P*_interaction_ = 0.047). Among male participants, the prevalence of prediabetes was higher among subjects in the first (lower) tertile (aPR_tertile 1 vs. tertile 3_ = 2.18, 95% CI: 1.35–3.52) and second (middle) tertile (aPR_tertile 2 vs. tertile 3_ = 2.24, 95% CI: 1.42–3.55) compared to subjects in the third (upper) tertile of vitamin D levels. Vitamin D tertiles were not associated with the prevalence of prediabetes among female participants (Table 4). Results of the sensitivity analyses (i.e., adjusting for VAT mass instead of BMI; online supplementary Table S2) did not differ from the primary analyses (Table 4).

**Table 4:** Adjusted associations between 25-hydroxyvitamin D levels and prediabetes stratified by sex.

	Males		Females		
25-hydroxyvitamin D (nmol/L)	aPR^a^ (95% CI)	*P*-value	aPR^a^ (95% CI)	*P*-value	*P*_interaction_^e^
**Model 1**^**b**^					
Per 10-unit decrease	1.19 (1.06–1.34)	0.004	1.02 (0.94–1.11)	0.584	0.032
**Model 2**^**c**^					
In-/sufficiency	1.00 (Reference)	–	1.00 (Reference)	–	0.026
Deficiency	2.35 (1.36–4.07)	0.002	1.03 (0.60–1.77)	0.927	
**Model 3**^**d**^					
Tertile 3	1.00 (Reference)	–	1.00 (Reference)	–	0.047
Tertile 2	2.24 (1.42–3.55)	<0.001	1.28 (0.67–2.45)	0.453	
Tertile 1	2.18 (1.35–3.52)	0.002	1.01 (0.49–2.06)	0.990	

*aPR* adjusted prevalence ratio, *CI* confidence interval.

^a^Adjusted for age, body mass index, smoking status, walking frequency, and season of blood collection.

^b^Prevalence ratio of prediabetes was estimated per 10-unit decrease in 25-hydroxyvitamin D (nmol/L). The 25-hydroxyvitamin D (nmol/L) variable was modeled as a continuous variable.

^c^Insufficiency/sufficiency was defined as: 25-hydroxyvitamin D ≥ 50 nmol/L. Deficiency was defined as: 25-hydroxyvitamin D < 50 nmol/L. The insufficiency/sufficiency category was the reference.

^d^The 25-hydroxyvitamin D (nmol/L) variable was categorized into tertiles, with the third tertile being the reference category.

^e^In the total analytical sample that included data of males and females, the interaction was assessed by including the following product term in the regression model: ‘sex × 25-hydroxyvitamin D status.’ In model 1, 25-hydroxyvitamin D variable was included in the model as a continuous variable. In model 2, 25-hydroxyvitamin D variable was modeled as a dichotomous variable. In model 3, 25-hydroxyvitamin D tertile variable was entered in the model as a categorical variable.

**Fig. 1:**
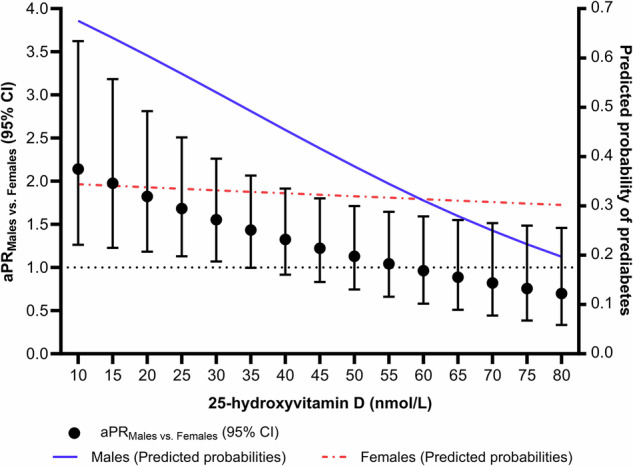
Adjusted prevalence ratios (aPRs) relating sex and prediabetes at different levels of 25-hydroxyvitamin D (nmol/L) levels (left y-axis), and adjusted predicted probabilities of prediabetes (right y-axis) across levels of 25-hyroxyvitamin D in male (solid blue line) and female (red dashed line) participants. The aPRs along with their 95% confidence intervals (CIs) were estimated while comparing the prevalence of prediabetes in males to the prevalence of prediabetes in females at different levels of 25-hydroxyvitamin D (nmol/L). The estimated aPRs and predicted probabilities were adjusted for age, body mass index, smoking status, walking frequency, and season of blood collection. The dotted horizontal line refers to the null value of “1”.

Moreover, to further understand the observed sex-specific association between vitamin D and prediabetes, we have tested the association between sex and prediabetes prevalence at different levels of 25(OH)D, and have estimated the predicted probabilities of prediabetes in male and female participants across levels of 25(OH)D (Fig. 1). Male compared to female participants had higher prevalence of prediabetes at 25(OH)D levels of ≤35 nmol/L. However, this sex-related difference was not apparent at 25(OH)D levels of >35 nmol/L (Fig. 1). The predicted probabilities of prediabetes among female participants were consistent across levels of 25(OH)D. However, among male participants, the predicted probabilities of prediabetes showed a decreasing pattern as 25(OH)D levels increased (Fig. 1).

## Discussion

In this study conducted among adults without diabetes, after controlling for confounding factors, we found that vitamin D levels were inversely associated with prediabetes prevalence among male participants, with deficient vitamin D levels being associated with increased prediabetes prevalence. Such an association was not observed among female participants, where vitamin D levels did not demonstrate an association with prediabetes prevalence. The prevalence of prediabetes was higher in male compared to female participants at 25(OH)D levels of ≤35 nmol/L. Whereas, at 25(OH)D levels of >35 nmol/L, there were no sex differences in the prevalence of prediabetes. These findings suggest that sex can be an effect modifier of the association between vitamin D and prediabetes.

Numerous studies carried out in different countries have reported sex differences in vitamin D levels, as summarized in a meta-analysis of 7.9 million individuals [37]. A survey conducted in the US on 15,804 individuals revealed that vitamin D deficiency was more common in men, however, an earlier study showed that women were actually more prone to vitamin D deficiency [38, 39]. Another study by Albuloshi et al. [40] conducted in Kuwait showed similar results to our study with vitamin D deficiency being slightly more common in male participants compared to female participants, which they explained by the fact that more women might be consuming vitamin D supplements compared to men [40]. Additionally, a study conducted in Saudi Arabia showed that women exhibit a lower general exposure to the sun due to the extremely hot weather conditions and their modest attire, however, their knowledge about the importance of vitamin D was greater than that in men, and they took vitamin D supplements more frequently than men [41]. A higher intake of vitamin D supplements might also explain why we observed an increase in 25(OH)D levels with advancing age, with a significant difference in women, but we cannot say that with certainty as no data was collected on vitamin D supplement intake in our study.

Multiple biological mechanisms explaining the observational associations between vitamin D and diabetes/prediabetes have been examined, with studies consistently showing that a vitamin D deficiency contributes to increased insulin resistance and altered pancreatic β-cell function, which can lead to reduced insulin secretion, and the development of diabetes [19, 20]. The proposed mechanisms by which vitamin D appears to be protective against diabetes include increasing insulin secretion, improving insulin sensitivity, and reducing systemic inflammation [42]. Vitamin D increases insulin secretion by binding to VDR, which is expressed in the pancreatic β-cells. Additionally, insulin production is a calcium-dependent process, and vitamin D regulates extracellular calcium concentrations and influx into the β-cells [42]. Vitamin D enhances insulin sensitivity by stimulating the production of insulin receptors. Moreover, Vitamin D contributes to the reduction of systemic inflammation through the down-regulation of transcription factors responsible for the production of pro-inflammatory cytokines [26].

Although the role of vitamin D supplementation in preventing or possibly delaying the progression from prediabetes to diabetes has been inconsistent in subjects with prediabetes, a meta-analysis of three randomized clinical trials showed that vitamin D supplementation compared to placebo was associated with a 15% (pooled hazard ratio [HR]: 0.85, 95% CI: 0.75–0.96) reduced risk of progressing to diabetes, with greater reduction in diabetes risk (HR: 0.24, 95% CI: 0.16–0.36) observed among subjects who maintained intra-trial 25(OH)D concentrations ≥125 nmol/L (≥50 ng/mL) [43]. Similarly, a meta-analysis demonstrated that vitamin D supplementation in persons with prediabetes reduced the progression to diabetes and increased the reversion to normoglycemia, with more benefits observed among subjects with normal weight [44]. Another meta-analysis among subjects with prediabetes, showed no overall improvements in insulin resistance and glycemic control in relation to vitamin D supplementation [45]. However, subgroup analysis showed that vitamin D supplementation improved insulin resistance among subjects with baseline 25(OH)D levels ≥50 nmol/l [45]. Moreover, secondary outcome analyses showed that vitamin D supplementation was associated with reduced HbA1c and fasting plasma glucose in subjects with prediabetes [45]. A prior study showed that subjects with prediabetes and deficient vitamin D levels compared to subjects with prediabetes and sufficient vitamin D levels had a higher degree of insulin resistance and reduced insulin secretory function, making this group more susceptible to the development of diabetes [46]. Therefore, the existing evidence supports the beneficial effect of vitamin D supplementation in persons with prediabetes, with these effects varying according to adiposity status and baseline/intra-trial 25(OH)D concentrations. Such evidence further strengthens the possible causal effect of vitamin D deficiency in prediabetes/diabetes development.

Observational studies have reported associations between vitamin D and prediabetes, with recent large meta-analyses showing that a vitamin D deficiency is associated with an increased risk of prediabetes [17, 18]. In terms of effect modification, contradictory results have been reported by a few studies, with some showing that the association between vitamin D and prediabetes/diabetes varies based on other variables, such as race/ethnicity, urban/rural setting, hypertension, alcohol use, and sex, while other studies did not find such heterogeneity in the effects [15, 47–50]. In the current report, deficient vitamin D levels were associated with an increased prevalence of prediabetes among male participants, but not among female participants. Moreover, prediabetes prevalence was increased in male compared to female participants at 25(OH)D levels of ≤35 nmol/L, with this sex-related difference diminishing at 25(OH)D levels of >35 nmol/L. Xu et al. showed possible effect modification by sex (p for interaction = 0.083) of the association between vitamin D and insulin resistance, with increased vitamin D levels being associated with reduced insulin resistance among males, but not among females. [47]. Moreover, Knekt et al. showed that increased vitamin D levels were associated with reduced incidence of diabetes among men; whereas among women, vitamin D levels were not associated with diabetes incidence [51]. Among individuals newly diagnosed with diabetes, a negative association between vitamin D levels and insulin resistance was reported only among male participants [52]. Similarly, an inverse association between vitamin D levels and insulin resistance among males with overweight was reported [53]. Among individuals with normal concentrations of glucose in the blood, decreased vitamin D levels were associated with increased fasting glucose and reduced insulin secretory function in males only [25]. In contrast, Stadlmayr et al. showed that low vitamin D levels were associated with increased diabetes risk in females, but not in males [54]. Chen et al. reported an inverse association between vitamin D levels and insulin resistance among females with vitamin D deficiency, but not among males [55]. Although some inconsistencies exist in the literature, overall, the aforementioned observational findings indicated that sex could modify the effect of vitamin D on prediabetes/diabetes risk and related biomarkers.

Hormonal differences play an important role in the sex-related differences among males and females with regards to susceptibility to health conditions such as diabetes [24, 56]. Our study showed increased prediabetes prevalence among males as 25(OH)D levels decreased and the lack of such an association in females. This could possibly be explained by the protective role of endogenous estrogen, which activates alpha receptors (ERα) in various bodily tissues. For example, estrogen has been shown to improve insulin-stimulated glucose uptake by the muscle and increase glucose-mediated insulin secretion by the pancreatic β-cells [56, 57]. When looking at the interaction between estrogen and vitamin D, studies have shown a synergistic relationship between the two, where vitamin D appears to stimulate the production of estrogen, and estrogen together with vitamin D can stimulate the expression of insulin receptors thereby improving blood glucose uptake by cells [56]. Moreover, we observed sex-related differences in the prevalence of prediabetes (higher in males than females) at 25(OH)D levels of ≤35 nmol/L, which further indicates that vitamin D is more potent in females than in males. For instance, in females, the predicted probabilities of prediabetes across levels of 25(OH)D were consistent (Fig. 1), implying that the effectiveness of vitamin D on prediabetes is attained at low and high levels of 25(OH)D in females. In contrast, among males, the predicated probabilities of prediabetes were high at lower levels of 25(OH)D, and these predicted probabilities decreased as 25(OH)D increased. Hence, males with low levels of 25(OH)D are more susceptible to prediabetes than those with higher 25(OH)D levels. Collectively, sex may influence vitamin D levels as well as modulate the effect of vitamin D on disease risk. Also important to note, is that the protective effect of female sex hormones (estrogen), tends to diminish with the onset of menopause [58]. Although our study did not ask women if they had transitioned into menopause, we did notice that the prevalence of prediabetes increased as age increased in both males and females even though vitamin D levels increased as age increased only in females. The increased prevalence of prediabetes in females was especially evident in females in the age groups of 45-54 and ≥55 years, which span the typical age of onset of menopause [59].

Another factor that might explain the observed sex differences in the association of vitamin D with prediabetes is the sexual dimorphism in fat distribution, with males accumulating more visceral fat (risk factor for metabolic diseases), and females storing more fat in the subcutaneous depot prior to menopause (protective of metabolic diseases) [57, 60]. Nonetheless, in our primary analysis, we controlled for BMI when assessing the association between vitamin D and prediabetes. Moreover, given that adiposity can be a major confounding factor of the measures of association relating vitamin D and disease risk [36], we conducted sensitivity analyses while adjusting for VAT mass instead of BMI (online supplementary Table S2). The results of the sensitivity analysis did not differ from the primary analysis (Table 4), hence confirming the robustness of the reported associations against confounding by the type of adiposity measure.

The population-based sample of adults without diabetes is a strength of our study. Although the current analysis was based on a subsample (*n* = 384) of the total sample (*n* = 1184), the characteristics of the subsample and total sample were similar. Hence, selection bias is less likely to have an effect on the reported results. Moreover, identifying statistically significant associations indicates that the analytical sample size provided sufficient statistical power. Serum 25(OH)D was not measured by the gold standard method of liquid chromatography tandem-mass spectrometry (LC-MS/MS), which could have led to some measurement error. However, we have modeled 25(OH)D levels using three strategies, as a continuous, dichotomous, and categorical variable, to ensure the consistency and validity of the results. All three modeling approaches provided similar findings, which indicates that misclassification, if any, did not majorly influence the reported results. A further limitation was the lack of information on potential confounders that include vitamin D supplement use, sun exposure, alcohol consumption, dietary intake of vitamin D and calcium-rich foods, hormone replacement therapy, menopausal status, and parathyroid hormone levels. The cross-sectional design of our study only allowed us to evaluate concurrent associations, and not potentially causal effects.

In conclusion, this study showed that vitamin D levels are associated with prediabetes in a sex-specific manner. In particular, deficient levels of vitamin D (25(OH)D < 50 nmol/L) were associated with increased prevalence of prediabetes among males, but not among females. Moreover, the difference between the sexes was observed at 25(OH)D levels of ≤35 nmol/L, with a higher prevalence of prediabetes in males compared to females. Such a sex-disparity was diminished at 25(OH)D levels of >35 nmol/L. Future studies should assess the association of vitamin D levels as well as the effectiveness of vitamin D supplementation on the risk of prediabetes/diabetes separately for males and females, as such stratification might provide a better assessment for risk stratification and aid targeted preventive interventions.

### Supplementary information

Supplementary information: Table S1 and Table S2

## Data Availability

The datasets analyzed during the current study are available from the corresponding author on reasonable request.
