# Supplementary material for: Sex differences in the association between vitamin D and prediabetes in adults: A cross-sectional study
Source: Nutr Diabetes. 2024 Jul 2;14:49. doi: 10.1038/s41387-024-00311-4 (PMC11219834; doi:10.1038/s41387-024-00311-4)
Supplement: Supplementary file 1 — Supplementary information: Table S1 and Table S2 [file 41387_2024_311_MOESM1_ESM.pdf]

Supplementary Information

Article title:

Sex differences in the association between vitamin D and prediabetes in adults: a cross-sectional study

Authors and Affiliations:

Ali H. Ziyab[1,*], Anwar Mohammad[2], Zainab Almousa[1], Talal Mohammad[3]

[1] Department of Community Medicine and Behavioral Sciences, College of Medicine, Kuwait University, Safat, Kuwait;

[2] Dasman Diabetes Institute, Biochemistry and Molecular Biology Department, Kuwait City, Kuwait;

[3] St. Antony's College, University of Oxford, Oxford, UK.

[*] **Corresponding author:**

Ali H. Ziyab, PhD

Department of Community Medicine and Behavioral Sciences

College of Medicine, Kuwait University

P. O. Box 24923, Safat 13110, Kuwait

Tel: (+965) 24636545

Fax: (+965) 25338948

E-mail: ali.ziyab@ku.edu.kw

Table S1. Characteristics of the total enrolled sample and the analytical sample

Variables	Total sample (n = 1184)	Analytical sample* (n = 384)
**Sex, % (n)**		
Male	49.4 (585)	55.7 (214)
Female	50.6 (599)	44.3 (170)
**Age (years), % (n)**		
Overall, Median (IQR)	44.0 (34.0-51.0)	40.5 (33.0-48.0)
≤34	26.2 (310)	30.5 (117)
35–44	28.0 (331)	32.0 (123)
45–54	30.3 (359)	27.1 (104)
≥55	15.5 (184)	10.4 (40)
**Cigarette smoking status, % (n)**		
Never smoker	61.0 (445)	54.5 (180)
Former smoker	15.5 (113)	17.6 (58)
Current smoker	23.5 (171)	27.9 (92)
Missing, (n)	(455)	(54)
**Walking in the past 4 weeks, % (n)**		
None	28.8 (123)	29.5 (100)
1 to 3 times in the last 4 weeks	36.8 (157)	36.0 (122)
1 to 3 times a week	22.0 (94)	21.8 (74)
4 or more times a week	12.4 (53)	12.7 (43)
Missing, (n)	(757)	(45)
**Prediabetes (HbA1c), % (n)**		
Yes (5.7–6.4%)	32.6 (355)	35.2 (135)
No (<5.7%)	67.4 (733)	64.8 (248)
Missing, (n)	(96)	(1)
**BMI (kg/m^2), % (n)**		
Underweight/normal weight (<25.0)	20.1 (238)	21.4 (82)
Overweight (25.0 to <30.0)	44.4 (526)	45.6 (175)
Obesity (≥30.0)	35.5 (420)	33.0 (127)
**VAT (kg), Median (IQR)**		
Overall (n = 1183†; 384‡)	0.89 (0.49-1.47)	0.89 (0.45-1.51)
Tertile 1 (n = 410†;137‡)	0.37 (0.21–0.55)	0.31 (0.18–0.49)
Tertile 2 (n = 403†;136‡)	0.90 (0.68–1.34)	1.08 (0.72–1.44)
Tertile 3 (n = 370†;111‡)	1.80 (1.24–2.20)	1.87 (1.32–2.29)
**25-hydroxyvitamine D (nmol/L), % (n)**		
Severe deficiency (<25)	–	34.9 (134)
Deficiency (≥25 to <50)	–	28.1 (108)
Insufficiency (≥50 to <75)	–	24.7 (95)
Sufficiency (≥75)	–	12.3 (47)
**25-hydroxyvitamine D (nmol/L), Median (IQR)**		
Overall (n = 384‡)	–	32.0 (20.0–62.0)
Tertile 1 (n = 123‡)	–	16.0 (14.0–20.0)
Tertile 2 (n = 137‡)	–	32.0 (26.0–55.0)
Tertile 3 (n = 124‡)	–	69.0 (53.5–82.5)

IQR: interquartile range; HbA1c: glycated hemoglobin; BMI: body mass index; VAT: visceral adipose tissue.
* Subsample (n = 384) with 25-hydroxyvitamine D measurement.
† Number of participants in the total study sample.
‡ Number of participants in the analytical sample.

Table S2 – Sensitivity Analysis. Adjusted associations between 25-hydroxyvitamin D levels and prediabetes stratified by sex

	Males		Females		
**25-hydroxyvitamin D (nmol/L)**	**aPR* (95% CI)**	***P*-value**	**aPR* (95% CI)**	***P*-value**	$P_{interaction}$⁋
**Model 1†**					
Per 10-unit decrease	1.17 (1.04-1.31)	0.007	1.02 (0.94-1.11)	0.622	0.041
**Model 2‡**					
In-/sufficiency	1.00 (Reference)	–	1.00 (Reference)	–	0.028
Deficiency	2.25 (1.31-3.88)	0.003	1.01 (0.60-1.70)	0.972	
**Model 3#**					
Tertile 3	1.00 (Reference)	–	1.00 (Reference)	–	0.049
Tertile 2	2.21 (1.41-3.48)	<0.001	1.27 (0.66-2.44)	0.472	
Tertile 1	1.93 (1.18-3.17)	0.009	0.99 (0.49-2.02)	0.983	

aPR: adjusted prevalence ratio; CI: confidence interval.

* Adjusted for age, visceral adipose tissue (VAT) mass, smoking status, walking frequency, and season of blood collection.

† Prevalence ratio of prediabetes was estimated per 10-unit decrease in 25-hydroxyvitamin D (nmol/L). The 25-hydroxyvitamin D (nmol/L) variable was modeled as a continuous variable.

‡ Insufficiency/sufficiency was defined as: 25-hydroxyvitamin D ≥ 50 nmol/L. Deficiency was defined as: 25-hydroxyvitamin D < 50 nmol/L. The insufficiency/sufficiency category was the reference.

\# The 25-hydroxyvitamin D (nmol/L) variable was categorized into tertiles, with the third tertile being the reference category.

⁋ In the total analytical sample that included data of males and females, interaction was assessed by including the following product term in the regression model: 'sex × 25-hydroxyvitamin D status.' In model 1, 25-hydroxyvitamin D variable was included in the model as a continuous variable. In model 2, 25-hydroxyvitamin D variable was modeled as a dichotomous variable. In model 3, 25-hydroxyvitamin D tertile variable was entered in the model as a categorical variable.
